# Simulation of Death in the East

**Published:** 1892-02

**Authors:** 


### SIMULATION OF DEATH IN THE EAST.

The powers of the fakirs, or faqueers, of India and Persia of simulating death are marvelous, and almost incredible. Several sects in these countries regard the art of apparent death as a part of their religious ritual, and practice it assiduously. In their ancient books it is described as puranayam, or stopping the breath. Many cases in which these Indian fakirs have allowed themselves to be buried alive for long periods have been verified by British officials in India, and attested by evidence which dispels all doubt of their truth. This personation of death continues for as long as six, and even ten months. The way the fakirs go to work to produce this condition, is to have the little ligature under the tongue cut, whereby they are enabled to stretch this organ out to a great length. Then they turn it back, inserting the end in the throat, and closing up at the same time the inner nasal apertures. The externa apertures of the nose and the ears are closed with wax, and the eyes covered to exclude the light. Long preliminary practice is, however, needed in holding the breath, and a long course of fasting before burial, The fakir then sinks into a condition resembling death, and the body is wrapped in linen, placed in a box and buried. When the box is taken up, at the expiration of the long continued death-like sleep, and opened. the fakir is found cold and stiff ; no pulsation can be felt ; the heart, the wrist, the temples are still ; the body is not cold as a corpse would be, but is colder than that of other living men, except over the seat of the brain. All the secretions are fully stopped, the nails, hair, and beard have ceased growth. After being resuscitated the fakir feels great dizziness, and for a few hours cannot stand up without support, but gradually recovers strength, and enjoys amazingly the wonder he has excited.

